# Lack of significant seasonal association between serum 25(OH)D concentration, muscle mass and strength in postmenopausal women from the D-FINES longitudinal study

**DOI:** 10.1017/jns.2022.106

**Published:** 2022-12-13

**Authors:** Anneka E. Welford, Andrea L. Darling, Sarah J. Allison, Susan A. Lanham-New, Carolyn A. Greig

**Affiliations:** 1 School of Sport, Exercise and Rehabilitation Sciences, University of Birmingham, Edgbaston, Birmingham B15 2TT, UK; 2 Leicester Diabetes Centre, University of Leicester, Leicester General Hospital, Leicester LE5 4PW, UK; 3 Nutrition, Food and Exercise Sciences Department, School of Biomedical Sciences, Faculty of Health and Medical Sciences, University of Surrey, Guildford GU2 7XH, UK; 4 MRC-Versus Arthritis Centre for Musculoskeletal Ageing Research, University of Birmingham, Edgbaston, Birmingham B15 2TT, UK; 5 NIHR Birmingham Biomedical Research Centre, University Hospitals Birmingham NHS Foundation Trust & University of Birmingham, Birmingham, UK

**Keywords:** Muscle mass, Muscle strength, Longitudinal, Sarcopenia, Seasonal variation, Vitamin D, 1,25(OH)2D, 1,25-dihydroxyvitamin D, 25(OH)D, 25-hydroxyvitamin D, ASM, appendicular skeletal mass, BMI, body mass index, D-FINES, Vitamin D, Food Intake, Nutrition and Exposure to Sunlight in Southern England, DEQAS, vitamin D quality assurance scheme, DXA, dual X-ray absorptiometry, EWGSOP, European Working Group on Sarcopenia in Older People, GP, General Practitioner, HGS, handgrip strength, kg, kilogram, ng/ml, nanograms per millilitre, nmol/l, nanomoles per litre, PAL, physical activity level, RASM, relative appendicular skeletal mass, SPSS, Statistical Package for Social Sciences, UK, United Kingdom, USA, United States of America, VDR, vitamin D receptor

## Abstract

The aim of the present study was to assess the seasonal relationship between serum 25(OH)D concentration, lean mass and muscle strength. This was a secondary data analysis of a subgroup of 102 postmenopausal women participating in the 2006–2007 D-FINES (Vitamin D, Food Intake, Nutrition and Exposure to Sunlight in Southern England) study. The cohort was assessed as two age subgroups: <65 years (*n*=80) and ≥65 years (*n*=22). Outcome measures included lean mass (DXA), muscle strength (handgrip dynamometry) and serum 25(OH)D concentration (enzymeimmunoassay). Derived outcomes included appendicular skeletal muscle mass (ASM) and relative appendicular skeletal muscle index (RASM). Sarcopenia status was assessed using the European Working Group on Sarcopenia in Older People 2018 criteria. Non-parametric partial correlation using BMI as a covariate was used to evaluate the study aims. There were no statistically significant associations between total lean mass, ASM or RASM and 25(OH)D in any group at any season. There was a trend for handgrip strength to be positively associated with serum 25(OH)D concentration. There was a trend showing a higher prevalence of sarcopenia in women ≥65 years. Sarcopenia status appeared transient for five women. In conclusion, the present study found no significant association between vitamin D status and functional indicators of musculoskeletal health, which were additionally not affected by season.

## Introduction

Vitamin D deficiency is highly prevalent and has been recently described as a ‘global pandemic’^(1)^. Older adults are particularly at risk^(2)^, likely a result of a decreased cutaneous capacity to synthesise vitamin D^(3)^, limited sun exposure due to physical inactivity and reduced time spent outdoors^(4)^. It is well established that serum 25-hydroxyvitamin D (25(OH)D) concentration varies seasonally^(5–8)^, although the variation may be minimal^(9)^ or absent^(10,11)^ in older adults.

The discovery of the vitamin D receptor (VDR) and the reduction in its expression in skeletal muscle tissue with age suggests a direct effect of vitamin D on muscle^(12)^. In an animal study, murine C2C12 cells treated with 25(OH)D and 1,25-dihydroxyvitamin D (1,25(OH)_2_D) increased VDR and CYP24A1 expression^(13)^. A downregulation of myostatin was also observed, supporting the theory that vitamin D has a direct effect on proliferation, differentiation and a potential anabolic effect on myotube size^(13)^. Additionally, case-reports, observational and epidemiological studies demonstrate that vitamin D deficiency is associated with myopathy and myalgia^(14)^, which are reversible upon correction of the deficiency^(14–16)^.

Age-related losses in muscle mass, strength and function, known assarcopenia^(17)^, are associated with poor quality of life^(18)^ and are prevalent in fallers and those at risk of falling^(19)^; individuals with combined low muscle mass and function are predicted to be 12⋅3 times more likely to lose their independence at 90 years of age^(20)^.

The role of vitamin D in the regulation of muscle mass and/or muscle strength is not well understood and data are presently limited. However, recent evidence using animal models has demonstrated that muscle atrophy is triggered by reduced VDR expression; *Tibialis anterior* myofibre area was decreased in rats with VDR-knockdown via a reduction in total protein content. Autophagy was identified to be the mechanism of atrophy, with an impairment to mitochondrial and myogenic function and related gene-set expression noted^(21)^. Further work reported by the same group concluded that mice over-expressing the VDR in the *Tibialis anterior* muscle demonstrated muscle hypertrophy^(22)^. These findings were later extrapolated to humans; following 20 weeks of resistance training, VDR expression was found to be a reliable marker of the hypertrophic response in healthy participants aged 18–75 years^(22)^.

Conflicting reports of a positive association between 25(OH)D and muscle mass^(23–26)^ and strength^(27–32)^ oppose studies reporting no association with muscle mass^(31–33)^ or strength^(33)^.

To our knowledge, there has been no investigation to determine if sarcopenia status is season-dependent. Serum 25(OH)D concentration is known to be affected by season^(34–36)^; as discussed earlier, it is possible that muscle mass and strength are associated with 25(OH)D concentration. If so, it is plausible to suggest that sarcopenia status, calculated at different seasonal timepoints throughout the year, may vary. Therefore, the primary aim of the present study was to analyse data obtained from a large cohort of postmenopausal women, investigating associations between serum 25(OH)D concentration and lean mass and strength, alongside the influence of season.

## Materials and methods

Data were analysed from the 2006–2007 D-FINES study (Vitamin D, Food Intake, Nutrition and Exposure to Sunlight in Southern England^(34)^); a cohort study of pre- and postmenopausal white British/Irish and British Asian women. We analysed data from *n*=102 postmenopausal women, comprising *n*=80 <65 years and *n*=22 ≥65 years.

The D-FINES study was conducted in accordance with the 1964 Declaration of Helsinki and received Research Ethics Committee approval (National Health Service NHS REC 06/Q1909/1 and University of Surrey EC/2006/19/SBMS). Written informed consent was obtained from all participants.

The D-FINES study is presented in detail elsewhere^(34)^. Briefly, 365 women were recruited from General Practitioner (GP) surgeries in the southeast of England. Exclusion criteria included conditions resulting in a disorder of calcium homeostasis, currently taking medications affecting bone, calcium or vitamin D metabolism, supplemental use of vitamin D or cod liver oil and abnormal liver, thyroid or renal function or use of hormone replacement therapy within the previous year. Participants were invited to the University of Surrey Clinical Investigation Unit on four occasions: during the summer (21 June 2006 and 20 September 2006), autumn (21 September 2006 and 20 December 2006), winter (21 December 2006 and 20 March 2007) and spring (21 April 2007 and 20 June 2007). Postmenopausal status was defined as not menstruating for over 3 months. During each visit, anthropometric data (height, weight, grip strength) and venous blood samples (25(OH)D) were collected. Additionally, during the autumn of 2006 and the spring of 2007, body composition was measured using dual X-ray absorptiometry (DXA).

### Serum 25(OH)D concentration measurement

Fasting venous blood samples were collected during each season. Samples were analysed by the Vitamin D Research Group laboratory based at the Manchester Royal Infirmary, which is certified by the International Organization for Standardization (ISO 9001:2000 and ISO 13485:2003) and participates in the Vitamin D quality assurance scheme (DEQAS). Serum 25(OH)D concentration was measured using a manual enzyme immunoassay (Immunodiagnostic Systems Ltd, Boldon, Tyne and Wear, UK). Manufacturer reference ranges for 25(OH)D were 19–58 ng/ml (48–144 nmol/l, with some seasonal variability), sensitivity was 2 ng/ml (5 nmol/l), intra- and inter-assay coefficients of variation were 6 and 7 %, respectively. Seasonal variation in serum 25(OH)D concentration has been reported within the D-FINES cohort^(7,34)^, however, subgroups of postmenopausal women, younger and older than 65 years have not.

### Muscle strength assessment

Handgrip strength (HGS) was assessed during the summer, autumn, winter and spring using a JAMAR® hydraulic hand dynamometer (J. A. Preston Corporation, Clifton, NJ, USA). The participant was seated, forearms resting on the arms of the chair with their wrists just over the end of the chair's arm and their feet flat on the floor for measurements. Comfort was assessed and the size of the handle adjusted if necessary. Mean maximal HGS, expressed in kilograms, was calculated from a set of three measurements on each hand (left and right).

### Lean mass assessment

Total body lean mass was assessed via DXA using a Hologic QDR 4500 (Hologic Inc, USA) in autumn 2006 and spring 2007. Mean difference in lean mass estimates between operators of +0⋅58 (0⋅47) kg and an interrater coefficient of variation of 1⋅9 % have been reported in 22 human cadavers (22 % female, mean age = 79⋅6 years, mean weight = 69⋅6 kg), repositioned and scanned three times^(37)^. DXA, computerised tomography^(38)^ and magnetic resonance imaging^(39)^ estimates of lean mass correlate highly. DXA is considered a reference standard for lean mass estimation^(40)^ recommended by the European Working Group on Sarcopenia in Older People, 2018 (EWGSOP2)^(17)^.

### Derived muscle outcomes

Appendicular skeletal muscle mass (ASM) was calculated as the sum of the lean mass of the arms and legs in kilograms. ASM was divided by height squared (relative appendicular skeletal muscle index – RASM); this variable is suggested as a method to quantify relative muscle mass by controlling for body size^(17,41–43)^. Sarcopenia was defined according to the EWGSOP2 2018 criteria for women^(17)^; low muscle strength (HGS of the right hand <16 kg) and low muscle mass (ASM <15 kg), assessed in autumn and spring. Sarcopenic status and subsequent analyses were calculated only for participants with ASM and HGS data available for both the seasons.

### Physical activity assessment

Physical activity level (PAL) was assessed via a questionnaire^(44)^ reporting estimates of the number of hours per day spent in sleep, light, moderate, and active work and lesiure time activity. PALs were calculated by using previously reported equations: light, 1⋅56; moderate, 1⋅64; active, 1⋅82^(45)^.

### Statistical analysis

IBM SPSS Statistics for Windows (Chicago, IL), version 26⋅0 was utilised, with statistical significance set at an α-level of *P* = 0⋅05 unless otherwise stated. Data deviated from the normal distribution; as log transformation was unable to normalise all outcome variables, data were maintained in their original form and non-parametric analyses were completed.

The relationship between seasonal total lean mass, ASM and 25(OH)D concentration was assessed using Spearman correlation. Non-parametric partial correlation using BMI (body mass index) as a covariate was used to assess associations between serum 25(OH)D concentration and RASM and muscle strength outcomes throughout the seasons. BMI has been utilised as a covariate in similar studies due to the known influence of BMI/body size on serum 25(OH)D concentration and strength^(17,27,46)^.

Differences between sarcopenic and non-sarcopenic participants were assessed using the Mann–Whitney *U* test. *χ*^2^ tests were used to analyse differences between groups in categorical variables at baseline. Fisher's exact test (2 × 2 contingency table) assessed associations between postmenopausal subgroup and sarcopenic status.

The cohort was analysed as three groups: the whole group and two subgroups; women <65 years and ≥65 years, as age is a major risk factor for sarcopenia^(47)^ and contributes to declining anabolic hormone concentration^(48)^, increasing physical inactivity^(49)^ and malnutrition^(50)^.

## Results

Data from 102 postmenopausal women were analysed, including 80 aged <65 years (mean age = 58⋅6 (3⋅2); age range = 48–64 years) and 22 aged ≥65 years (mean age = 67⋅5 (1⋅7); age range = 65–71 years; Table 1). The cohort were overweight (defined as BMI = 25⋅0 to <30) and there were no significant differences in characteristics, except for age, between the groups. There was a trend for lower HGS at all seasonal timepoints for women aged ≥65 years.

**Table 1.** D-FINES study participant characteristics

	All postmenopausal women (*n* = 102)	Postmenopausal women <65 years (*n* = 80)	Postmenopausal women ≥65 years (*n* = 22)	
	Mean (sd)	Mean (sd)	Mean (sd)	*P*
Age (years)	60⋅51 (4⋅67)	58⋅60 (3⋅17)	67⋅45 (1⋅68)	<0⋅001
Height (cm)	160⋅50 (6⋅16)	160⋅64 (6⋅61)	159⋅98 (4⋅22)	0⋅569
Ethnicity (%)	87% White, 11% South Asian, 2% Other	84% White, 15% South Asian, 2% Other	100% White, 0% South Asian, 0% Other	0⋅122
Autumn weight (kg)	68⋅62 (13⋅44)	68⋅57 (13⋅85)	68⋅80 (12⋅10)	0⋅939
Autumn BMI (kg/m^2^)	26⋅69 (5⋅26)	26⋅64 (5⋅48)	26⋅86 (4⋅52)	0⋅868
Autumn total lean mass (kg)	39⋅61 (5⋅31)	39⋅93 (5⋅39)	39⋅33 (4⋅52)	0⋅538
Autumn total fat mass (kg)	25⋅24 (8⋅68)	25⋅32 (9⋅02)	24⋅99 (7⋅47)	0⋅883
Spring weight (kg)	68⋅73 (13⋅54)	68⋅81 (14⋅09)	68⋅41 (11⋅62)	0⋅891
Spring BMI (kg/m^2^)	26⋅73 (5⋅28)	26⋅74 (5⋅89)	26⋅71 (4⋅37)	0⋅982
Spring total lean mass (kg)	39⋅86 (5⋅38)	39⋅78 (5⋅53)	40⋅17 (4⋅91)	0⋅765
Spring total fat mass (kg)	25⋅25 (8⋅79)	29⋅40 (9⋅16)	24⋅70 (7⋅46)	0⋅744
Summer (HGS)	21⋅82 (4⋅81)	22⋅19 (4⋅85)	20⋅46 (4⋅55)	0⋅135
Autumn (HGS)	21⋅91 (4⋅60)	22⋅27 (4⋅57)	20⋅59 (4⋅54)	0⋅135
Winter (HGS)	22⋅10 (4⋅77)	22⋅53 (4⋅84)	20⋅51 (4⋅24)	0⋅078
Spring (HGS)	21⋅73 (5⋅09)	22⋅08 (5⋅31)	20⋅49 (4⋅06)	0⋅196

BMI, body mass index; HGS, handgrip strength.

Mean serum 25(OH)D concentration and corresponding vitamin D status by season are presented in Table 2. Briefly, mean serum 25(OH)D concentration ranged from 39⋅2 to 61⋅5 nmol/l and 0–37⋅5 % of the cohort were vitamin D deficient, varying by season. Serum 25(OH)D concentrations were highest and the lowest percentage of participants were vitamin D deficient in the summer.

**Table 2.** Seasonal 25(OH)D concentrations and corresponding vitamin D status of all postmenopausal women and women <65 years and ≥65 years subgroup

	Postmenopausal women aged <65 years				Postmenopausal women aged ≥65 years			
	Mean 25(OH)D (sd)	Adequate (%)	Insufficient (%)	Deficient (%)	Mean 25(OH)D (sd)	Adequate (%)	Insufficient (%)	Deficient (%)
Summer	56⋅6 (20⋅7)	62⋅5	26⋅3	11⋅3	61⋅5 (19⋅7)	68⋅2	31⋅8	0⋅0
Autumn	50⋅4 (20⋅2)	46⋅3	37⋅5	16⋅3	52⋅3 (16⋅1)	45⋅45	54⋅55	0⋅0
Winter	39⋅2 (16⋅5)	26⋅3	36⋅3	37⋅5	40⋅1 (14⋅2)	22⋅7	50⋅0	27⋅3
Spring	42⋅7 (19⋅5)	32⋅5	37⋅5	30⋅0	41⋅5 (18⋅7)	22⋅7	54⋅5	22⋅7

Vitamin D adequacy defined as serum 25(OH)D concentrations ≥50⋅0 nmol/l, inadequacy defined as 30⋅0–49⋅9 nmol/l and deficiency defined as ≤29⋅9 nmol/l.

No significant interaction between age group and serum 25(OH)D concentration (*F*(3) = 1⋅83, *P* = 0⋅250).

As with premenopausal women in the D-FINES cohort^(34)^, postmenopausal British Asian women presented with significantly lower 25(OH)D concentrations than White British/Irish women and could be classified as vitamin D deficient during each season (summer: 28⋅41 nmol/l *v*. 61⋅64 nmol/l, autumn: 21⋅45 nmol/l *v*. 55⋅30 nmol/l, winter: 21⋅11 nmol/l *v*. 41⋅91 nmol/l, spring: 22⋅63 nmol/l *v*. 45⋅23 nmol/l, all *P* < 0⋅001).

Overall, the cohort had sufficient serum 25(OH)D concentrations during the summer (57⋅67 (20⋅47) nmol/l) and autumn (50⋅81 (19⋅31) nmol/l), but were insufficient during the winter (39⋅40 (15⋅96) nmol/l) and spring (42⋅67 (18⋅66) nmol/l). Women <65 years had the lowest mean serum 25(OH)D concentration (in the winter) and women ≥65 years had the highest mean serum 25(OH)D concentration (in the summer).

### Serum 25(OH)D concentration and lean mass and strength

There were no statistically significant associations between serum 25(OH)D and any variables displayed in Table 3. Unadjusted analyses are displayed as Appendices (Supplementary Appendix A and B). After adjustment for BMI, there was a trend for a negative association between RASM and 25(OH)D concentration in all groups at all timepoints. This trend was also observable for ASM, except for postmenopausal women aged ≥65 years. Within the whole cohort, there was no association between change in serum 25(OH)D from autumn and spring and the change in total lean mass.

**Table 3.** Associations between serum 25(OH)D concentration, lean mass and muscle strength within the D-FINES cohort

	Whole cohort (*n* 102)		Postmenopausal women aged <65 years (*n* 80)		Postmenopausal women aged ≥65 years (*n* 22)	
	*r*_s_	*P*	*r*_s_	*P*	*r*_s_	*P*
	(95 % CI)		(95 % CI)		(95 % CI)	
25(OH)D × total lean mass Autumn	−0⋅053 (−0⋅3, 0⋅2)	0⋅598	−0⋅032 (−0⋅3, 0⋅2)	0⋅775	0⋅020 (−0⋅4, 0⋅5)	0⋅928
25(OH)D × total lean mass Spring	−0⋅017 (−0⋅2, 0⋅3)	0⋅866	−0⋅019 (−0⋅2, 0⋅2)	0⋅864	0⋅137 (−0⋅4, 0⋅6)	0⋅543
25(OH)D × ASM Autumn	−0⋅185 (−0⋅4, 0⋅1)	0⋅062	−0⋅169 (−0⋅4, 0⋅1)	0⋅134	−0⋅077 (−0⋅6, 0⋅5)	0⋅734
25(OH)D × ASM Spring	−0⋅145 (−0⋅3, 0⋅1)	0⋅145	−0⋅169 (−0⋅4, 0⋅1)	0⋅134	0⋅085 (−0⋅4, 0⋅5)	0⋅706
25(OH)D × Relative appendicular skeletal muscle index Autumn*	−0⋅225 (−0⋅4, −0⋅6)	0⋅026	−0⋅243 (−0⋅4, −0⋅2)	0⋅031	−0⋅217 (−0⋅6, 0⋅2)	0⋅344
25(OH)D × Relative appendicular skeletal muscle index Spring*	−0⋅182 (−0⋅4, 0⋅1)	0⋅069	−0⋅180 (−0⋅4, −0⋅2)	0⋅113	−0⋅160 (−0⋅6, 0⋅2)	0⋅487
Change in total lean mass × change in 25(OH)D Autumn to Spring	−0⋅130 (−0⋅3, 0⋅1)	0⋅194	−0⋅103 (−0⋅3, 0⋅1)	0⋅361	−0⋅316 (−0⋅6, 0⋅1)	0⋅152
25(OH)D × HGS Summer*	0⋅103 (0⋅1, 0⋅4)	0⋅103	0⋅138 (−0⋅1, 0⋅3)	0⋅226	0⋅059 (−0⋅4, 0⋅4)	0⋅798
25(OH)D × HGS Autumn*	0⋅182 (0⋅1, 0⋅4)	0⋅026	0⋅096 (−0⋅3, 0⋅1)	0⋅400	−0⋅139 (−0⋅7, 0⋅3)	0⋅548
25(OH)D × HGS Winter*	0⋅132 (−0⋅1, 0⋅3)	0⋅187	0⋅165 (−0⋅1, 0⋅3)	0⋅145	0⋅023 (−0⋅5, 0⋅4)	0⋅920
25(OH)D × HGS Spring*	0⋅125 (−0⋅1, 0⋅3)	0⋅212	0⋅116 (−0⋅1, 0⋅3)	0⋅308	0⋅120 (−0⋅5, 0⋅6)	0⋅605

ASM, appendicular skeletal muscle mass; RASM, relative appendicular skeletal muscle mass index is ASM/h^2^; HGS, handgrip strength.

* Model is adjusted for BMI; all other variables are unadjusted.

*P* relates to Spearman correlation analysis and significance is set at *P* < 0⋅02 following Bonferroni correction for multiple comparisons.

There was a trend for a positive association between HGS and 25(OH)D concentration within all participant groups at all timepoints, following adjustment for seasonal BMI. The exception was for women ≥65 years during the autumn, for whom statistical significance was not observed following the application of Bonferroni corrections for multiple comparisons.

Additionally, there was no association between serum 25(OH)D concentration and lean mass and muscle strength when assessing by vitamin D status, with the exception of ASM, which was significantly and positively associated with 25(OH)D concentrations for vitamin D deficient participants in the spring (Table 4).

**Table 4.** Associations between serum 25(OH)D concentration and lean mass and muscle strength according to vitamin D status

	Sufficient 25(OH)D concentration			Insufficient 25(OH)D concentration			Deficient 25(OH)D concentration		
	*n*	*r*_s_ (95 % CI)	*P*	*N*	*r*_s_ (95 % CI)	*P*	*N*	*r*_s_ (95 % CI)	*P*
25(OH)D × ASM Autumn	47	−0⋅003 (−0⋅3, 0⋅3)	0⋅984	42	−0⋅010 (−0⋅3, 0⋅3)	0⋅950	13	0⋅362 (−0⋅2,0⋅9)	0⋅248
25(OH)D × ASM Spring	31	0⋅123 (−0⋅2, 0⋅4)	0⋅518	43	0⋅048 (−0⋅3, 0⋅3)	0⋅765	28	0⋅382 (−0⋅1, 0⋅7)	0⋅049
25(OH)D × HGS Summer*	65	0⋅008 (−0⋅2, 0⋅3)	0⋅953	28	−0⋅107 (−0⋅5, 0⋅3)	0⋅595	9	0⋅431 (−0⋅3, 0⋅9)	0⋅287
25(OH)D × HGS Autumn*	47	0⋅142 (−0⋅9, 0⋅4)	0⋅348	42	0⋅107 (−0⋅2, 0⋅4)	0⋅505	13	0⋅123 (−0⋅6, 0⋅8)	0⋅703
25(OH)D × HGS Winter*	26	0⋅080 (−0⋅3, 0⋅6)	0⋅704	40	0⋅147 (−0⋅2, 0⋅5)	0⋅370	36	0⋅165 (−0⋅1, 0⋅5)	0⋅344
25(OH)D × HGS Spring*	31	0⋅052 (−0⋅3, 0⋅6)	0⋅785	43	0⋅192 (−0⋅1, 0⋅5)	0⋅223	28	−0⋅060 (−0⋅4, 0⋅4)	0⋅767

* Model is adjusted for BMI; all other variables are unadjusted.

ASM, appendicular skeletal muscle mass; HGS, handgrip strength.

Vitamin D adequacy defined as serum 25(OH)D concentrations ≥50⋅0 nmol/l, inadequacy defined as 30⋅0–49⋅9 nmol/l and deficiency defines as ≤29⋅9 nmol/l nmol/l.

*P* relates to the Spearman Correlation analysis.

### Seasonal sarcopenia classification

The percentage of women classified as sarcopenic was highest in the spring in all groups (Table 5). Sarcopenic status appeared transient for five women. Fisher's exact test highlighted no significant associations between age group and sarcopenic status during any season.

**Table 5.** Seasonal sarcopenia classification

	*N* classified as sarcopenic in Autumn (%)	*P*	*N* classified as sarcopenic in Spring (%)	*P*	Sarcopenic in both Autumn and Spring (%)	*P*	Sarcopenic only in Autumn (%)	*P*	Sarcopenic only in Spring (%)	*P*
Whole cohort (*n* 102)	4 (3⋅9)		7 (6⋅9)		3 (2⋅9)		1 (1⋅0)		4 (3⋅9)	
Postmenopausal women aged <65 years (*n* 80)	2 (2⋅5)	0⋅203	4 (5⋅0)	0⋅169	1 (1⋅3)	0⋅117	1 (1⋅3)	1⋅000	3 (3⋅8)	1⋅000
Postmenopausal women aged ≥65 years (*n* 22)	2 (9⋅9)		3 (13⋅6)		2 (9⋅1)		0 (0⋅0)		1 (4⋅6)	

Sarcopenia classification based on the EWGSOP2 criteria of combined low muscle mass and strength, which for women is handgrip strength <16 kg and ASM <15 kg^(47)^.

Included participants have all data required available during the autumn and spring timepoints.

Fisher's exact test was used to calculate *P*-values. Significance was set at *P* < 0⋅05.

25(OH)D concentrations of sarcopenic women were persistantly lower than the non-sarcopenic women; summer 46⋅90 (23⋅41) nmol/l *v*. 58⋅11 (20⋅36) nmol/l; autumn 45⋅56 (20⋅88) *v*. 51⋅03 (19⋅33) nmol/l; winter 32⋅20 (6⋅99) nmol/l *v*. 39⋅70 (16⋅17) nmol/l and spring 30⋅90 (9⋅48) nmol/l *v*. 42⋅94 (18⋅81) nmol/l for sarcopenic *v*. non-sarcopenic women, respectively. A similar trend for 25(OH)D concentration was observed for sarcopenic women in the spring compared with non-sarcopenic women; summer 52⋅91 (19⋅21) nmol/l *v*. 58⋅02 (20⋅21) nmol/l; autumn 47⋅94 (17⋅03) *v*. 51⋅02 (19⋅53) nmol/l; winter 33⋅13 (16⋅51) nmol/l *v*. 39⋅87 (15⋅91) nmol/l and spring 37⋅41 (14⋅92) nmol/l *v*. 42⋅84 (18⋅92) nmol/l for sarcopenic *v*. non-sarcopenic women, respectively.

Four women were classified as sarcopenic in the autumn; one woman was vitamin D deficient, one was insufficient and two were sufficient during the autumn. Seven women were classified as sarcopenic in the spring; two were vitamin D deficient, four were insufficient and one woman was sufficient. Of women who were sarcopenic during the autumn and spring, two were vitamin D sufficient in the autumn and insufficient in the spring. The other woman found to be sarcopenic during both seasons was vitamin D insufficient during the autumn and deficient during the spring.

## Discussion

The D-FINES cohort provided a unique opportunity to assess skeletal muscle health and function and their association with serum 25(OH)D. Serum 25(OH)D concentrations followed the anticipated seasonally dependent pattern, with peak concentrations observed during the summer and lowest concentrations observed during the winter and spring.

Serum 25(OH)D concentration was highest within postmenopausal women ≥65 years at all seasonal timepoints. One explanation for this finding is that the women <65 years group included a higher percentage of British Asian women (14 % *v*. 0 and 84 % *v*. 100 %, respectively); these women presented with significantly lower mean 25(OH)D concentrations than White British/Irish women at all seasonal timepoints. Vitamin D deficiency prevalence was higher than reported elsewhere, with the exception of postmenopausal women ≥65 years in the summer and autumn; 10 % of the total adult population (50–105 years) in England have been reported as vitamin D deficient^(51)^.

Women ≥65 years exhibited lower HGS than those <65 years at all seasonal timepoints, which is consistent with longitudinal reports of age-related decline in muscle strength^(52)^. HGS of both subgroups fell within the 25th centile for corresponding age group normative HGS data^(53)^. British Asian women were weaker than White British/Irish women at all seasonal timepoints, statistically evident during the winter and spring.

Clinical muscle weakness was not indicated during any season (HGS <16 kg^(17,54)^), however, individual-level data revealed muscle weakness present in 8⋅8–12⋅5 % of women <65 years and 9⋅1–18⋅2 % ≥65 years, varying with season (most apparent in the spring). Low muscle quantity (EWGSOP2 – ASM <15⋅00 kg)^(17)^ was not indicated, however, individual-level data revealed 37⋅5–41⋅3 % of women <65 years and 27⋅3–31⋅8 % ≥65 years had low muscle quantity, varying seasonally (most apparent in the spring).

No significant association between serum 25(OH)D concentration and total lean body mass, ASM or RASM was observed in any group or season. This is consistent with previous studies^(32,33,55)^, including those adjusting for season^(33,55)^. The trend towards a positive association between serum 25(OH)D concentration and muscle strength is consistent with previous studies^(27,28–32)^.

Muscle strength varies seasonally; in a study of 88 adults (mean age = 69⋅2 years), ankle dorsiflexion strength peaked in the summer, although was not significantly associated with 25(OH)D concentration^(6)^. 25(OH)D concentration and HGS has been shown to vary seasonally; a significant association was observable in the summer, but not winter in a group of 51 COPD (Chronic Obstructive Pulmonary Disease) patients (mean age = 68⋅7 years)^(32)^.

In our study, the lack of a statistically significant association between serum 25(OH)D concentration and skeletal muscle mass, but a positive trend with muscle strength suggests that vitamin D influences muscle strength and/or functioning rather than hypertrophy. A longitudinal study demonstrated that muscle mass explained only 5 % of the decline in strength, significant only for knee flexor strength in women^(52)^. Furthermore, physical activity is known to be associated with sarcopenia and vary seasonally^(56)^, with outdoor exercise positively influencing cutaneous production of vitamin D^(57)^. The average self-reported daily walking time varied seasonally within the D-FINES cohort, with a trend towards more time spent walking in the summer. It is possible that the seasonal physical activity variation may be driving the changes in muscle outcomes, although it was not possible to demonstrate this.

Sarcopenia prevalence within the D-FINES cohort (range = 2⋅5–9⋅1 %), was consistent with previous reports^(58)^, trending towards being highest in the spring within women ≥65 years; this was anticipated since sarcopenia prevalence is known to increase with age^(59)^.

ASM, total lean mass and HGS were significantly lower in sarcopenic participants than non-sarcopenic participants in the autumn and spring (all *P* < 0⋅005, data not shown). There was a non-significant trend for serum 25(OH)D concentration being lower and less variable in sarcopenic participants throughout the seasons, concurring with previous data demonstrating 25(OH)D concentration as significantly lower during the summer and lower, albeit not significantly, throughout the winter (*P* = 0⋅017) in sarcopenic *v*. non-sarcopenic adults^(60)^.

The sample size of women >65 years (*n* 22) and the subgroup of sarcopenic women (*n* 4 in autumn and *n* 7 in spring) within this cohort was small, meaning that the results found may not be generalisable to the general population of the UK and should be interpreted with caution. Furthermore, the results analysed are from a cohort assessed in 2006; a number of variables have changed, including the population demographics of the UK (the overall population has increased, as has the number of people aged over 65 years)^(61)^ and the way that ethnicity data are collected and coded^(62)^.

However, a novel finding of the present study was that sarcopenia status may be transient and season-dependent, although this was concluded from a small number of participants (four in autumn and seven in spring). Participants classified as sarcopenic based on the EWGSOP2 criteria^(17)^ differed from autumn to spring, with the highest percentage of sarcopenic women identified in the spring for both groups. Five women had a transient sarcopenic status, with four of the five women (80 %) sarcopenic only in the spring. Three of the four women <65 years (75 %) were sarcopenic only in spring and all postmenopausal women ≥65 years with transient sarcopenic status were observed in spring.

HGS may not present a true reflection of total body strength, since upper body strength loss is slower than within the lower body; knee extensor strength has been shown to decline by an additional 5⋅6 % per decade than elbow flexion strength^(52)^. Furthermore, HGS is vulnerable to positioning, particularly the elbow^(63)^. Finally, lower extremity muscle power may be preferable when assessing sarcopenic status, since muscle power is a superior predictor of functional status than muscle strength^(64,65)^ and more relevant to activities of daily living such as stair climbing, walking and standing from a seated position^(66)^.

## Conclusion

The present study found no association between serum 25(OH)D concentration and total lean mass, ASM or RASM at any season. There was a non-significant trend for muscle strength to be positively associated with 25(OH)D at all seasonal timepoints (in comparison to the trend for a negative relationship observed with muscle mass outcomes), suggesting that serum 25(OH)D concentration may be associated with muscle strength and functional outcomes rather than muscle hypertrophy. Sarcopenic status was shown to be transient and seasonal in a small sample of postmenopausal women; confirmation of this finding in a larger, more representative sample including men would allow more confidence in this outcome.

## Supplementary material

For supplementary material accompanying this paper visit https://doi.org/10.1017/jns.2022.106. click here to view supplementary material
