# Supplementary material for: Lack of significant seasonal association between serum 25(OH)D concentration, muscle mass and strength in postmenopausal women from the D-FINES longitudinal study
Source: J Nutr Sci. 2022 Dec 13;11:e107. doi: 10.1017/jns.2022.106 (PMC9794962; doi:10.1017/jns.2022.106)
Supplement: Supplementary file 1 [file S2048679022001069sup001.docx]

**Lack of significant seasonal association between serum 25(OH)D concentration, muscle mass and strength in postmenopausal women from the D-FINES longitudinal study.**

**Contents:**

**Appendix A:** Unadjusted associations between serum 25(OH)D concentration, lean mass and muscle strength according to postmenopausal age group.**……………………………………………………………………………2**

**Appendix B:** Unadjusted associations between serum 25(OH)D concentration and muscle strength according to vitamin D status**…………………………………………………………………………….3**

| **Appendix A: Unadjusted associations between serum 25(OH)D concentration, lean mass and muscle strength according to postmenopausal age group** | | | | | | |
| --- | --- | --- | --- | --- | --- | --- |
|  | **Whole cohort**  **(n = 102)** | | **Postmenopausal women aged <65 years**  **(n = 80)** | | **Postmenopausal women aged ≥65 years**  **(n = 22)** | |
|  | **r_s_ (95% CI)** | ***p*** | **r_s_ (95% CI)** | ***p*** | **r_s_ (95% CI)** | ***p*** |
| **25(OH)D x Relative appendicular skeletal muscle index Autumn*** | -.373 (-0.5, -0.2) | **<0.001** | -.395 (-0.6, -0.2) | **<0.001** | -.210 (-0.6, 0.3) | 0.349 |
|  | | | | | | |
| **25(OH)D x Relative appendicular skeletal muscle index Spring*** | -.335 (-0.5, -0.2) | **0.001** | -.381 (-0.6, -0.2) | **<0.001** | -.013 (-0.4, 04) | 0.954 |
|  | | | | | | |
| **25(OH)D X HGS Summer*** | .152 (-0.1, 0.4) | 0.127 | .176 (-0.1, 0.4) | 0.119 | .056 (-0.4, 0.5) | 0.804 |
|  | | | | | | |
| **25(OH)D X HGS Autumn*** | .039 (-0.2, 0.2) | 0.697 | .120 (-0.1, 0.4) | 0.289 | -.333 (-0.7, 0.1) | 0.130 |
|  | | | | | | |
| **25(OH)D X HGS Winter*** | .141 (-0.1, 0.3) | 0.157 | .186 (-0.1, 0.4) | 0.099 | -.085 (-0.6, 0.3) | 0.707 |
|  | | | | | | |
| **25(OH)D X HGS Spring*** | .193 (0.1, 0.4) | 0.052 | .227 (0.1, 0.4) | **0.043** | -.064 (-0.6, 0.4) | 0.778 |
| ASM: Appendicular skeletal muscle mass; Relative appendicular skeletal muscle mass is ASM/h^2^; HGS Handgrip strength.  *p* relates to the Spearman Correlation analysis | | | | | | |

| **Appendix B:** **Unadjusted associations between serum 25(OH)D concentration and muscle strength according to vitamin D status** | | | | | | | | | |
| --- | --- | --- | --- | --- | --- | --- | --- | --- | --- |
|  | **Sufficient 25(OH)D concentrations** | | | **Insufficient 25(OH)D concentrations** | | | **Deficient 25(OH)D concentrations** | | |
|  | **n** | **r_s_ (95% CI)** | ***p*** | **n** | **r_s_ (95% CI)** | ***p*** | **n** | **r_s_ (95% CI)** | ***p*** |
| **25(OH)D x HGS Summer** | 65 | -.024 (-0.3, 0.2) | 0.848 | 28 | .032 (-0.4, 0.5) | 0.870 | 9 | .167 (0.8, 0.8) | 0.668 |
|  | | | | | | | | | |
| **25(OH)D x HGS Summer** | 47 | .160 (-0.1, 0.5) | 0.282 | 42 | -.038 (-0.4, 0.3) | 0.813 | 13 | -.014 (-0.6, 0.5) | 0.964 |
|  | | | | | | | | | |
| **25(OH)D x HGS Summer** | 26 | .052 (-0.4, 0.5) | 0.799 | 40 | .100 (0.3, 0.5) | 0.540 | 36 | .092 (-0.2, 0.4) | 0.594 |
|  | | | | | | | | | |
| **25(OH)D x HGS Summer** | 31 | .229 (-0.2, 0.6) | 0.215 | 43 | .255 (-0.1, 0.5) | 0.098 | 28 | -.005 (-0.4, 0.4) | 0.982 |
| HGS Handgrip strength.  Vitamin D adequacy defined as serum 25(OH)D concentrations ≥50.00 nmol/L, insufficiency defined as 30.00 – 49.99 nmol/L and deficiency defined as ≤29.99 nmol/L.  *p* relates to the Spearman Correlation analysis. | | | | | | | | | |
